# Uridine‐responsive epileptic encephalopathy due to inherited variants in *CAD*: A Tale of Two Siblings

**DOI:** 10.1002/acn3.51272

**Published:** 2021-01-26

**Authors:** Christopher M. McGraw, Sonal Mahida, Parul Jayakar, Hyun Yong Koh, Alan Taylor, Trevor Resnick, Lance Rodan, Marc A. Schwartz, Ayesha Ejaz, Vijay G. Sankaran, Gerard Berry, Annapurna Poduri

**Affiliations:** ^1^ Epilepsy Massachusetts General Hospital Boston Massachusetts USA; ^2^ Department of Neurology Harvard Medical School Boston Massachusetts USA; ^3^ Epilepsy Genetics Program Boston Children’s Hospital Boston Massachusetts USA; ^4^ Division of Genetics and Metabolism Nicklaus Children’s Hospital Miami Florida USA; ^5^ Al Jalila Children’s Hospital Dubai United Arab Emirates; ^6^ Division of Pediatric Neurology Miami Children’s Hospital Miami Florida USA; ^7^ Division of Genetics and Genomics Boston Children’s Hospital Boston Massachusetts USA; ^8^ Division of Hematology/Oncology Boston Children’s Hospital Boston Massachusetts USA; ^9^ Department of Pediatrics Harvard Medical School Boston Massachusetts USA; ^10^ Department of Pediatric Oncology Dana Farber Cancer Institute Boston Massachusetts USA; ^11^ Broad Institute of MIT and Harvard Cambridge Massachusetts USA

## Abstract

We report two siblings with intractable epilepsy, developmental regression, and progressive cerebellar atrophy due to biallelic variants in the gene *CAD*. For the affected girl, uridine started at age 5 resulted in dramatic improvements in seizure control and development, cessation of cerebellar atrophy, and resolution of hematological abnormalities. Her older brother had a more severe course and only modest response to uridine started at 14 years old. Treatment of this progressive condition via uridine supplementation provides an example of precision diagnosis and treatment using clear outcome measures and biomarkers to monitor efficacy.

## Introduction

The *CAD* gene encodes the multifunctional enzyme carbamoyl phosphate synthetase 2, aspartate transcarbamylase, and dihydroorotase 2 (abbreviated CAD), which plays a critical role in the *de novo* pyrimidine synthesis pathway.^1^ Biallelic CAD variants cause a severe neurometabolic disorder associated with developmental delay and epilepsy (known as CAD deficiency, or uridine‐responsive epileptic encephalopathy). Additional features include abnormal gait, progressive cerebellar atrophy, and hematologic abnormalities.^1^, ^2^ Features of CAD deficiency partly overlap with those of congenital disorders of glycosylation (CDG), but conventional CDG studies are reportedly normal.^1^, ^2^ Whole exome sequencing is currently the primary means of identifying CAD deficiency.

Here we report two siblings with novel biallelic *CAD* variants, with differences in phenotypic severity, timing of genetic diagnosis, and response to uridine. We demonstrate outcomes and biomarkers relevant to CAD deficiency and examine the spatiotemporal expression of *CAD* as it relates to key features of this disorder.

## Case presentations

### Patient 1 (proband)

**Figure 1:**
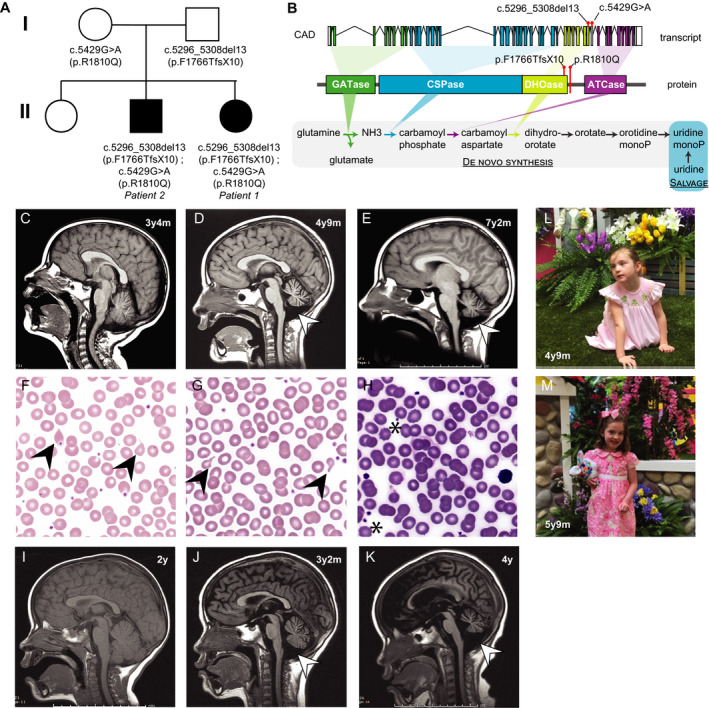
Identification of CAD variants, neuroimaging findings, and response to uridine. (A) Pedigree of CAD variants and disease status. Circle denotes female, square denotes male, and darkened symbols denote affected status. (B) Schematic of CAD gene transcript (*upper*), CAD protein domain architecture (*middle*) and key reactions in the pyrimidine synthesis pathway (*lower*). The location of the reported variants in exon 33 (involving DHOase domain) and exon 34 (in a linker region just beyond the DHOase domain) are shown as red lollipops (*upper* and *middle*). The first four reactions of the *de novo* pyrimidine synthesis pathway are catalyzed by enzymes encoded by CAD including glutamine aminotransferase (GATase), carbamoyl phosphate synthase (CSPase), aspartate transcarbamoylase (ATCase) and dihydroorotase (DHOase). Additional reactions to generate uridine monophosphate (uridine monoP) are abbreviated. A salvage pathway (*blue rectangle*) allows exogenous uridine to contribute to uridine monoP. (C‐E) and (I‐K), Neuroimaging from Patients 1 (C‐E) and 2 (I‐K), demonstrating more severe findings in Patient 2 (J vs C, and K vs D) for year of life, and stabilization of Patient 1 (D vs E) after initiating uridine at age 5. Images are sagittal T1‐weighted magnetic resonance imaging (MRI). (F–H) Blood smears demonstrate anisopoikilocytosis with occasional tear drop cells (*black arrowheads*) at 4 years (F) and 5 years (G) that normalize after uridine treatment at 7 years (H). Acanthocytes (*asterisks*, H) are believed to be artefactual. (L‐M) Image of Patient 1 prior to uridine treatment (L) and after 9 months of treatment (M).

Patient 1, a girl, is the third child born to nonconsanguineous parents (Fig. 1A). She has an affected older brother (Patient 2, below) and healthy older sister.

Patient 1 had delayed fine motor and language development, normal gross motor development, and a diagnosis of autism spectrum disorder at 2 years. Seizures began at 3.5 years with generalized tonic‐clonic (GTC) seizures. She later developed myoclonic seizures, focal motor seizures with secondarily generalized tonic‐clonic activity and postictal paresis (3‐4/week), and frequent atonic and absence seizures (up to 100/day). After a prolonged seizure at 4 years 8 months, she displayed low tone, could no longer ambulate independently, and plateaued in language skills. She had poor growth with head circumference 103 cm (−0.69 standard deviation [SD] for age) and weight 15.6 kg (−0.88 SD) at age 4 years 9 months. Trials of antiseizure medications (ASMs) did not reduce seizure frequency.

Brain MRI, normal at 3 years 4 months, showed mild cerebellar atrophy by 4 years 9 months (Fig. 1C‐D). Serum testing for CDG, including N‐glycan testing and transferrin isoelectric focusing, was normal. Urinary orotic acid and urinary pyrimidine panel were normal. Initial laboratory testing revealed mild macrocytosis without anemia, with red blood cell distribution at the upper limit of normal. Blood smear, evaluated after identification of *CAD* variants, showed moderate anisocytosis and poikilocytosis (**Fig. F‐G**).

### Patient 2

Patient 2 is the older brother of Patient 1 (Fig. 1A). He presented with delayed language and normal gross motor development. At 2 years, he had an onset of intractable GTCs (0‐2 per day). Atonic seizures began at 3 years. An episode of febrile status epilepticus (SE) at 3.5 years led to a 6‐month hospitalization that included pentobarbital coma, focal temporal lobe resection, and ventriculoperitoneal shunt placement. Subsequently, he showed substantial deterioration and remains nonverbal and tracheostomy‐dependent. Seizure control remained poor despite multiple ASM trials.

Brain MRI from 2 years old was normal (Fig. 1I); cerebellar atrophy was present at 3.5 years old (Fig. 1J). MRI at 4 years showed prominent hydrocephalus and ex vacuo changes with supratentorial and cerebellar atrophy (Fig. 1K). Routine laboratory testing was unremarkable, including normal CBC (Table 1).

**Table 1:** Clinical details of Patients 1 and 2

	Patient 1, F	Patient 2, M
Early developmental abnormalities	Delayed fine motor and language; esotropia; poor growth	Delayed language; esotropia
*Epilepsy history*		
Onset	3.5 years	2 years
Seizure types (frequency)	GTCS, myoclonic, focal motor seizures with postictal paresis (3‐4/wk); atonic and absence (100s/day)	GTCS (5‐10/day), atonic, status epilepticus
*Electroencephalographic (EEG) abnormalities*	Diffuse background slowing, generalized spike‐and‐slow wave (4y)	Disorganized background, focal onset seizures, tonic seizures correlating with paroxysmal fast activity
*Hematologic testing at presentation*		
MCV (range, 75‐87 fL)	Elevated (94.9 fL)	Reported normal
Hemoglobin (range, 11.5–15.5 g/dL)	Normal (13.1 g/dL)	Reported normal
RDW (range, 11.5‐15)	Upper limit of normal (14.7%)	Reported normal
Blood smear	Moderate anisocytosis, poikilocytosis	Not assessed
*CDG testing*	Normal	Not assessed
*MRI abnormalities*	Mild cerebellar atrophy (4 years, 9 months)	Cerebellar atrophy (3.5 years)
Uridine supplementation		
Initiation	5 years	14 years
Development	Motor activity, weight/HC ‐‐ normalized	Eye contact and expression ‐‐ improved
Seizures	GTCS/myoclonic/focal seizures – resolved.Absence seizures ‐‐ improved (few per day)	GTCS ‐‐ improved (0‐3 daily)
EEG	Improved background organization, ongoing focal sharps and generalized spike‐and‐slow‐wave discharges	Not assessed
Blood smear	Normalized	Not assessed
MRI	Stabilized	Stabilized

#### Genetic testing and identification of *CAD* variants as disease‐relevant

Whole exome sequencing revealed biallelic variants in *CAD* in both siblings (Table 2). The paternally inherited heterozygous c.5296_5308del13 deletion in *CAD* causes a 13‐base pair deletion resulting in a frameshift in the DHOase domain and a prematurely truncated protein lacking the last 455 of 2225 amino acids; it is predicted pathogenic by American College of Medical Genetics (ACMG) criteria.^3^ The maternally inherited heterozygous c.5429G > A missense variant causes an arginine to glutamine substitution within the DHOase domain (**Fig A, B**) and is classified as likely pathogenic.

**Table 2:** Variants identified in Patients 1 and 2. (Scores predicting pathogenicity are indicated as follows: green is predicted benign, red predicted deleterious, and orange uncertain.)

	Gene	Variant	Effect on protein	Reference db SNP	Prediction	Allele frequency in heterozygotes (gnomAD)	Allele frequency in homozygotes (gnomAD)	ClinVar	CADD	REVEL	MetaLR	Mutation Assessor	SIFT	Polyphen
1	*CAD*	c.5296_5308del13	p.Phe1766TfsX10	rs1019263242	pathogenic	6.4x10^‐5^	0	+	n/a	n/a	n/a	n/a	n/a	n/a
2	*CAD*	c.5429G > A	p.Arg1747Gln	rs139332887	likely pathogenic	3.52x10^‐4^	0	+	23	0.46	0.854	0.706	0.01	0.478
3	*GRIN2A*	c.2859G > C	p.E953D	rs749810424	VUS	7x10^‐56^	0	‐	9	0.66	0.009	0.022	1	0
4	*KIF26A*	c.5494G > A	p.E1832K	rs756446886	VUS	6.8x10^‐5^	0	‐	27	0.463	0.635	0.54	0	0.955
5	*KIF26A*	c.1643C > T	p.P548L	rs201175395	VUS	4.5x10^‐4^	0	‐	24	0.54	0.561	0.739	0	0.997

The phenotype of Patients 1 and 2 is consistent with that reported in association with *CAD*.^1^, ^2^, ^4^, ^5^ An unbiased serum metabolomic screen (Global Metabolomic Assisted Pathway Screen, Baylor Genetics) later demonstrated significantly reduced orotic acid (−3.4, Z‐score) and orotidine (−2, Z‐score) in Patient 1, consistent with disruption in the pyrimidine synthesis pathway (Fig. 1B), providing additional biochemical confirmation of CAD deficiency.

The patients also share variants of uncertain significance (VUS) in genes not known to be associated with their phenotype (Table 2). Chromosomal microarray was normal.

#### Spatiotemporal expression profile of CAD in adult and developing humans

**Figure 2:**
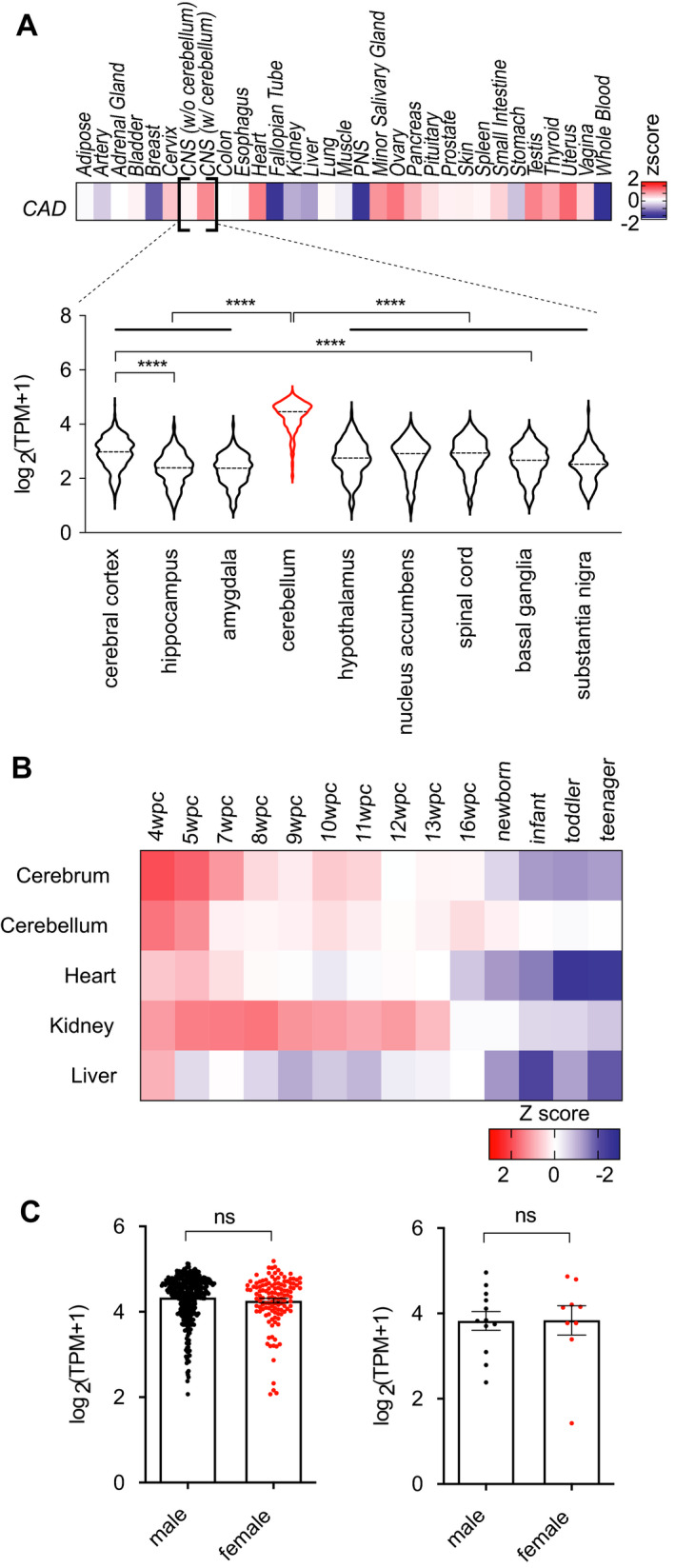
Spatiotemporal profiles of CAD expression in adult and developing humans. (A) CAD expression in adults. (*upper*) Heatmap of z‐scored normalized RNA‐seq expression estimates from the GTEx Project computed with respect to log_2_(TPM + 1) across 31 tissue types. (*lower*) Violin plots of normalized RNA‐seq expression estimates (transcripts per kilobase million plus one; log scale) by brain region. ****, adjusted p‐value < 0.0001. Tukey's multiple comparison test. (B) CAD expression in developing humans. Heatmap of z‐scored normalized RNA‐seq expression estimates computed with respect to log_2_ (TPM + 1) across five tissue types assayed at 14 time‐points^7^. *wpc*, weeks postconception. (C) No significant differences in CAD expression by sex in adult (*left*) or developing (*right*) cerebellum. Student’s t‐test, two‐tailed

To better understand the role of CAD function in the brain, we surveyed the spatiotemporal expression profile of *CAD* using publicly available human RNA sequencing data (Fig. 2; Supplemental Methods).

In adults, *CAD* expression is highest in sex‐specific tissues and peripheral nervous system (Z‐score, 1.0) and lowest in heart, muscle, and whole blood (Fig. 2A). In the central nervous system (CNS), *CAD* expression is highest in cerebellum (Z‐score, 0.6), with levels in the top 30th percentile (9th of 31) of queried tissues. All other queried regions of CNS have low *CAD* expression (overall Z‐score, −1.5; bottom 10th percentile, 28th of 31).

In developing humans, *CAD* expression in five tissues (cerebrum, cerebellum, heart, kidney, liver) is highest during early embryogenesis (Fig. 2B) and gradually falls over time. Its highest level overall is in cerebrum at 4 weeks postconception (w.p.c.) (Z‐score, 1.97; top 97th percentile across all tissues and timepoints). Postnatally, *CAD* expression in cerebrum falls over time (Z‐score range, −0.59 to −1.34), while expression in the cerebellum falls less drastically, becoming more highly expressed than in the cerebrum (Z‐score range, −0.11 to 0.21), suggesting sustained function in cerebellum compared to other organs. We did not observe sex‐specific differences in *CAD* expression in the cerebellum (Fig. 2C).

#### Patients’ responses to uridine treatment

After genetic diagnosis, we initiated oral uridine supplementation (off‐label), which has been used safely for CAD^1^, ^5^ and related metabolic conditions.^6^

#### Patient 1

Oral uridine (100mg/kg/day) was initiated at 5 years old. Within 6 months, gross motor development improved dramatically (Fig. 1M). She could run, somersault, and by age 7 swim independently. Her cognitive and language abilities improved; she recognizes letters and speaks short phrases. Fine motor skills also improved. Weight normalized: at 7 years 3 months she weighed 25kg (+0.53 SD).

Seizure control markedly improved. She had a single GTC soon after starting uridine and none since. At 8 years old, she remains free of GTCs and atonic seizures. Nondisabling absence seizures became less frequent but still occur daily. Clobazam and rufinamide had been added coincident with uridine; no further modifications to her regimen have been required. Uridine was increased step‐wise, guided in part by initial incomplete resolution of blood smear abnormalities, to 2700mg daily (~108 mg/kg/day). Blood smear now shows resolution of anisocytosis and poikilocytosis (Fig. 1H). Brain MRI at 7 years 2 months (Fig. 1E) demonstrated stabilization of infratentorial atrophy with no change in a 2.5‐year interval.

#### Patient 2

Oral uridine (100 mg/kg/day) was initiated at 14 years old. Seizure frequency decreased from 5‐10 daily GTCs to 0‐3 daily GTCs. Baseline health improved, with fewer infections and hospitalizations. He remains nonverbal and 100% dependent on care but is more engaged with family with regard to eye contact and emotional expression.

## Discussion

Our report of dramatic response to uridine treatment in Patient 1 demonstrates that CAD deficiency is treatable, providing the longest follow‐up reported to date (3 years). Earlier studies of patients with CAD deficiency have reported improvement up to 7 months following oral uridine supplementation^1^, ^5^, while two additional reports^2^, ^4^ do not provide details regarding the uridine response.

Despite sharing the same variants in *CAD*, Patient 2 presented with earlier and more severe seizures, earlier cerebellar atrophy (Fig. 1C,J), and more advanced cerebral and cerebellar atrophy (Fig. 1D,K) compared to his sister. Another study also reports a more severe male phenotype between two siblings.^1^ At the same time, late oral uridine supplementation in Patient 2 has resulted in less improvement than in Patient 1, suggesting that early diagnosis leading to early treatment is more effective.

The spatiotemporal profile of *CAD* expression suggests a role for CAD function in cerebellar development and function, which may have implications for disease pathophysiology. The gait dysfunction and cerebellar degeneration in patients with CAD deficiency may relate to a high demand for CAD in cerebellar tissue. How CAD function of the cerebellum relates to epilepsy in these patients, if at all, remains a mystery. There is no evidence of a sex‐specific difference in *CAD* expression to explain the observed male–female difference in severity between siblings with shared *CAD* variants.

We demonstrate that seizure frequency, developmental progress, MRI stabilization, and RBC normalization are appropriate measures for trials of uridine therapy for CAD deficiency. We recommend documentation of seizure frequency and formal developmental assessment at regular intervals, and MRI and blood smear evaluation to monitor disease stabilization and biochemical response to treatment. Our report underscores the urgency for CAD deficiency to be included in early diagnostic efforts, such as newborn screening. Abnormally low serum orotic acid and orotidine may be specific markers of CAD deficiency, particularly in patients whose presentations suggest CDG with negative biochemical testing, or in affected patients whose genetic work‐up reveals biallelic variants of uncertain significance in *CAD*. Given its imminently treatable nature, CAD deficiency should be considered early in the evaluation of epilepsy and developmental delay with regression.

## Conflicts of Interest

None to report.

## Authors’ contributions

CMM, SM, and AP drafted the manuscript. HYK performed expression analysis. All authors provided data analysis and interpretation and contributed to the editing of the manuscript.

## Supporting information

Supplementary Material
