# Supplementary material for: Uridine‐responsive epileptic encephalopathy due to inherited variants in CAD: A Tale of Two Siblings
Source: Ann Clin Transl Neurol. 2021 Jan 26;8(3):716–22. doi: 10.1002/acn3.51272 (PMC7951104; doi:10.1002/acn3.51272)
Supplement: Supplementary file 1 — Supplementary Material [file ACN3-8-716-s001.docx]

**Supplementary methods.**

**Gene expression analysis**

We analyzed tissue-specific *CAD* expression in healthy adults using Genotype-Tissue Expression (GTEx) RNA-seq data ([www.gtexportal.org](https://www.gtexportal.org/), accessed 6/1/2020, male n=331, female n=125), and during development using published RNA-seq data^8^ (ArrayExpress, accession no. E-MTAB-6814, accessed 6/1/2020) using R.
